# Analysis of SARS-CoV-2 genomic epidemiology reveals disease transmission coupled to variant emergence and allelic variation

**DOI:** 10.1038/s41598-021-86265-4

**Published:** 2021-04-01

**Authors:** D J Darwin R. Bandoy, Bart C. Weimer

**Affiliations:** 1 School of Veterinary Medicine, Population Health and Reproduction, 100K Pathogen Genome Project, University of California Davis, Davis, CA 95616 USA; 2 Department of Veterinary Paraclinical Sciences, College of Veterinary Medicine, University of the Philippines Los Baños, 4031 Los Baños, Laguna Philippines

**Keywords:** Computational biology and bioinformatics, Evolution, Genetics, Microbiology, Systems biology, Diseases, Medical research, Pathogenesis, Bioinformatics, Genomic analysis, Microbiology techniques

## Abstract

The spread of SARS-CoV-2 created a pandemic crisis with > 150,000 cumulative cases in > 65 countries within a few months. The reproductive number (R) is a metric to estimate the transmission of a pathogen during an outbreak. Preliminary published estimates were based on the initial outbreak in China. Whole genome sequences (WGS) analysis found mutational variations in the viral genome; however, previous comparisons failed to show a direct relationship between viral genome diversity, transmission, and the epidemic severity. COVID-19 incidences from different countries were modeled over the epidemic curve. Estimates of the instantaneous R (Wallinga and Teunis method) with a short and standard serial interval were done. WGS were used to determine the populations genomic variation and that underpinned creation of the pathogen genome identity (GENI) score, which was merged with the outbreak curve in four distinct phases. Inference of transmission time was based on a mutation rate of 2 mutations/month. R estimates revealed differences in the transmission and variable infection dynamics between and within outbreak progression for each country examined. Outside China, our R estimates observed propagating dynamics indicating that other countries were poised to move to the takeoff and exponential stages. Population density and local temperatures had no clear relationship to the outbreak progression. Integration of incidence data with the GENI score directly predicted increases in cases as the genome variation increased that led to new variants. Integrating the outbreak curve, dynamic R, and SNP variation found a direct association between increasing cases and transmission genome evolution. By defining the epidemic curve into four stages and integrating the instantaneous country-specific R with the GENI score, we directly connected changes in individual outbreaks based on changes in the virus genome via SNPs. This resulted in the ability to forecast potential increases in cases as well as mutations that may defeat PCR screening and the infection process. By using instantaneous R estimations and WGS, outbreak dynamics were defined to be linked to viral mutations, indicating that WGS, as a surveillance tool, is required to predict shifts in each outbreak that will provide actionable decision making information. Integrating epidemiology with genome sequencing and modeling allows for evidence-based disease outbreak tracking with predictive therapeutically valuable insights in near real time.

## Background

COVID-19 has reached global spread in all continents, except Antarctica, and was defined to be a pandemic by the World Health Organization (WHO) in March 2020^1–3^. As expected, outbreak dynamics are different among countries and regions. In part, this is due to environmental factors, contact networks, socio-cultural practices, human population characteristics, healthcare systems, the testing rate, and the public health strategies that include testing and surveillance strategies. The universal global response of face coverings, social distancing, and lockdowns has mitigated the spread temporarily but when these measures are lifted or ignored the outbreak quickly resumes.

Outbreaks are defined by the reproductive number (R)^4, 5^, a common measure of transmission for infectious disease spread. The probability of increased disease spread is evaluated based on the threshold when R > 1; conversely a decline in spread is observed with R < 1. Additionally, R can be used to estimate the proportion of the population that needs to be vaccinated in order to generate herd immunity^6^, as has been discussed in some countries as a method to control the pandemic and as a method to measure how well population immunity is occurring in absence of a vaccine. Use of R in the context of viral mutation has yet to be examined but likely has potential to be a valuable combinatorial approach once the framework for integration is defined.

Use of R for the 2020 COVID-19 pandemic was done for the initial outbreak in China as an estimate of the local epidemic expansion with the earliest estimates of R = 2.2 (95% CI 1.4 to 3.9) based on 424 cases in Wuhan, China^7^. Subsequent calculation of R, with 2033 cases from China (nationwide), slightly changed the estimate of R = 2.2 to 3.6^8^. However, estimates of R for other countries were not done routinely but rather a fixed estimate R was used based on the refined estimate based on the outbreak in China. However, even the refined estimate was inadequate in capturing spread dynamics of the pandemic and expansion within individual locations, indicating that R is not constant at different locations and that a more dynamic calculation is warranted. Use of static R estimates during the epidemic spread is underestimating location and population specific outbreak dynamics during local spread^4, 5^, which is currently in the 2nd and 3rd wave of spread. Hence, there is a need to rapidly estimate dynamic R values during the epidemic so as to better estimate potential local hot spots that will have rapid and unexpected increases in cases. This approach can also be useful to provide global comparisons of outbreak expansion at each global location that will enable public health responses to align with the epidemiological approaches across countries and locally.

The Wallinga and Teunis method for R estimation requires input of outbreak incidences and the period between the manifestation of symptoms in the primary case and the onset of symptoms in secondary cases to be the serial interval^9^. This approach was previously implemented in a web resource to estimate R during epidemics^10^. A key advantage of using dynamic estimates is the ease of estimating credible serial intervals, compared to other maximum likelihood estimation approaches that quickly provides valuable information to control spread of the outbreak. Additionally, integration of viral genetic variation with R estimates will provide additional information about changes in cases and indicate a change in risk. While there is seemingly no obvious relationship between R, severity of the epidemic, SARS-CoV-2 genome diversity^11^, the continual mutation of the viral genome makes this comparison an important and logical consideration to describe outbreak dynamics. If such an association exists appropriate interventions can be considered in specific locations rather than blanket mandates that negatively affect economic status of specific regions when they have little risk of disease spread. As the number of WGS continue to be generated, it is becoming clear that genome variation has a role in changing the epidemiological dynamics of the outbreak.

In spite of no clear path for systematic integration of viral genome evolution via SNP determination with epidemiology beyond lineage designations, the COVID-19 pandemic is demonstrating a global unity for sharing SARS-CoV-2 whole genome sequences (WGS) with unprecedented openness. By quickly sharing genome sequences it enables investigation of the genome variation using multiple approaches to sample the virial genome space that define changes that may lead to alteration of the outbreak dynamics. However, use of lineages to predict the outbreak is meeting challenges with small sample size^12, 13^ and independent mutations that lead to variant emerging in multiple locations globally. WGS availability is continuing to expand and has reached a number of WGS that constitutes as a viral population for analysis, which provides additional information that cannot be gleaned from a few sequences. Population genome analysis is particularly important for SARS-CoV-2 because of the high mutation rate, which was linked by estimating transmission dynamics of rapidly evolving RNA viruses. WGS integration highlights the opportunity to infer transmission by incorporating WGS into the outbreak progression and mitigation strategies^14, 15^. This approach was validated in Ebola virus (EBOV) and Middle East respiratory syndrome coronavirus (MERS-CoV) outbreaks where each virus is separated by a small number of mutations, yet these small changes produce new infection dynamics during respective outbreaks^16, 17^. Rapidly evolving pathogens undergo genome sequence mutation, random drift, local selection pressure, and stochastic variation that produce genomic versions of the viral genomes that is likely associated with new infections^14^, has been observed with the emergence of the B1.1.7 lineage and is currently fueling the 3^rd^ outbreak wave in the UK. Even small changes in the genome result in transmission changes that are determined by mutations between individual genomes and can be detected using WGS. SARS-CoV-2 genomes are changing over the course of outbreak but there is controversy about the impact and specifics mutations that lead to public health impacts and transmission dynamics. Viral mutations and the need for fast differentiation of changes highlights the value of systematically combining WGS with epidemiology.

Considering the lack of containment of the pandemic globally, except in Singapore, Hong Kong, and Taiwan, we hypothesized that the estimated basic R value for China do not provide reliable estimates for other countries. This is demonstrated by the observation that varies greatly by the time and location of the outbreak—highlighting the dynamic nature of R in outbreaks but more importantly in pandemics. The empirical observations of varying epidemiological curves by country, viral mutation rate, and geographically unique variation seem to accompany new cases around the world. These intertwined factors are likely individual mechanisms of change in sustaining the outbreak expansion of the pandemic. While viral sequencing is occurring quickly and the data are being made public, it is not being effectively integrated with epidemiological information because there is not an existing framework to systematically merge these different data streams. In this study, we used incidence data to estimate R and compared country specific COVID-19 transmission dynamics with viral population genome diversity. By incorporating R, the epidemic curve, and SARS-CoV-2 genome diversity we created a systematic framework that deduced how viral genome diversity can be used to describe epidemiological features of an outbreak before new cases were observed. This was done by creating a genome diversity metric that provides genome diversity context and allowed quantification of the infection dynamics globally that were divergent from the early estimates with genomic evidence. We call this approach the pathogen genome identity (GENI) scoring system. GENI scores, in combination with distinct outbreak stages, were indicative of new cases and found unrecognized local transmission.

## Methods

Chinese CDC and WHO situations reports were used to assemble the incidence data as compiled by the Center for Systems Science and Engineering by the John Hopkins University (Baltimore, MD, USA) that was accessed on March 1, 2020^18^ to construct epidemic curves (epicurves). We defined four groups along the epicurve that characterized increasing expansion and a decline phase that was used as markers of specific events for each outbreak.

The extracted time series case data were used as input for determining the instantaneous R on a daily basis to effectively capture dynamic changes in case reports. The estimates of R were selected at 2 and 7 days to examine fluctuations in reporting as between the defined phases. A parametric of uncertainty (offset gamma) and distributional estimates for the serial interval were used. A mean of 2 and 7 days, with standard deviation of 1 was used to capture short and standard serial interval assumptions using 50 sub-samples of the serial interval distribution. The Wallinga and Teunis method, as implemented by Ferguson^10^, is a likelihood-based estimation procedure that captures the temporal pattern of the effective R from an observed epidemic curve. R was calculated using the web application EpiEstim App (https://shiny.dide.imperial.ac.uk/epiestim/)^10^. The descriptive statistics were used to compute the mean and confidence intervals to estimate the instantaneous R.

The GENI score was anchored on the principle of rapid pathogen evolution between transmission events. This required defining a reference sequence from the outbreak, which in this study was the Wuhan seafood market pneumonia virus isolate designated as Wuhan-Hu-1 NC_045512.2^19^. Publicly available raw WGS were retrieved from GISAID (supplementary Table 1) and Nextstrain (supplementary Table 2) with appropriate metadata. Whole genome SNP variants were determination using Snippy (version 4.6.0)^20–22^ with the default settings (--mincov 10, --minqual 100, --cpus 32) using Wuhan-Hu-1 (Genbank MN908947.3) as the reference genome. The average mutation/isolate was divided by the total epicurve time (days) to derive a daily epidemic mutation rate that was scaled to a monthly rate that was produced. The transformed value of this rate was derived before integrating it with epidemiological information. The output from the variant calling step was then used to determine a GENI score by calculating the individual nucleotide difference over the entire genome from the reference. The basis for GENI score cutoffs, to estimate transmission dates, were derived from accepted evolutionary inference of mutation rates of SARS-CoV-2 of 2 mutations/month^23, 24^.

Four epicurve stages were identified to provide a clear method to define increases in the outbreak. First, the ‘index stage’ was characterized by the first report (index case) or limited local transmission indicated by intermittent zero incidence from an undulating epicurve. A second distinct stage was defined to be the ‘takeoff stage’, wherein the troughs were approximately the same level as the previous peak but no longer reached zero. Third, the ‘exponential stage’ was characterized by a sharp upward increase where the outbreak expanded quickly and a large number of new cases emerged daily. The last stage was defined as the ‘decline’ and was noted when the outbreak past the peak and newly reported case counts were smaller than the previous day. Transition into the decline stage ultimately resulted in few to no new cases being reported, yet viral circulation was still occurring and new WGS were being found in each outbreak.

## Results

**Figure 1:**
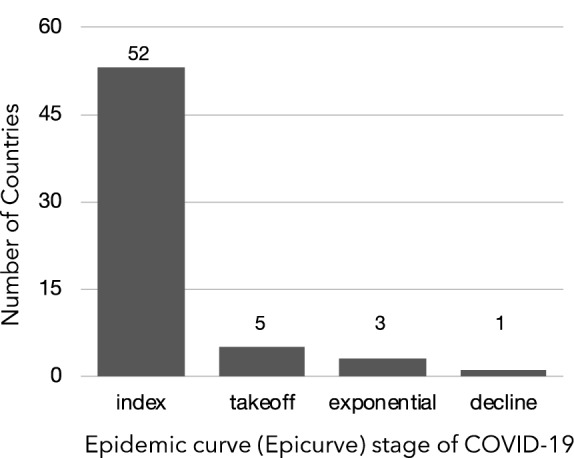
Distribution of country classification based on SARS-CoV-2 epicurve status.

The mutation rate calculations for SARS-CoV-2, based on the Wuhan reference genome, found the nucleotide change per month to be 1.7 (95% CI 1.4–2.0), similar to other estimates^11^, with substitutions occurring at 0.9 × 10^–3^ (95% CI 0.5–1.4 × 10^–3^) substitutions per site per year. This provided confidence that the reference genome was adequate for this study, so we proceeded to determine the outbreak dynamics of COVID-19 pandemic by classifying each country’s status according to epicurve stage with a framework of stages: (a) index (b) takeoff (c) exponential (d) decline as a clear method that can be used to benchmark metrics that allow a consistent integration of R and viral genome diversity measurement. First, R was determined using the instantaneous method with two different serial intervals—2 and 7 days (Table 1). As of March 1, 2020, this framework defined global epicurves as gaining momentum globally with 52 countries in the index stage. Three countries were in the exponential stage and five countries in the takeoff stage (Fig. 1). China was the only country that reached the peak of the epicurve and was characterized to be in the decline stage. No evidence of any other country near the decline stage, and some countries were poised to move into the takeoff and exponential phase based on the epicurve alone was observed.

**Table 1:** Country-specific instantaneous reproductive number (R) estimates for SARS-CoV-2 as of March 1, 2020.

Country	Cases	Instantaneous reproductive number (R) serial intervals	
		2 days	7 days
Mainland China	79,251	1.6	2.1
South Korea	3150	2.8	25.6
Italy	1128	8	57.0
Iran	593	2.8	17.1
Japan	241	3.6	2.2
Singapore	102	3.3	1.6
France	100	2.9	16.9
Hong Kong	95	2.6	1.6
Germany	79	3.1	17.2
United States	70	4.3	1.7
Kuwait	45	2.6	15.3
Spain	45	3.7	10.8
Thailand	42	3.8	1.7

**Figure 2:**
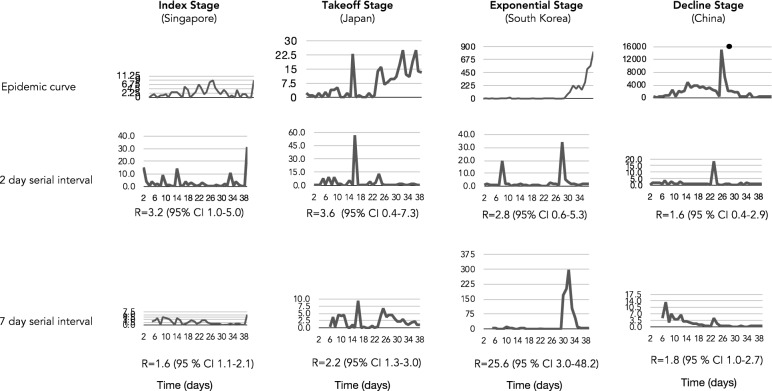
Instantaneous reproductive number estimates for different stages of the SARS-CoV-2 epidemic curve: **a** index (Singapore), **b** takeoff (Japan), **c** exponential (South Korea), **d** decline (China) in short (2 days) and standard (7 days) serial interval. Decelerating stage of epidemic curve results to a reproductive number lower than 2 for both serial intervals, epidemic curve with multiple introductions yields 2-day serial interval with higher reproductive number and exponential serial interval yields higher reproductive number for the 7-day serial interval. The surge in the epidemic curve of China corresponds to the alteration of the case definition of SARS-CoV-2 by broadening confirmed cases with pneumonia confirmed with a computed tomography scan. South Korea’s higher reproductive number is due to cryptic transmission associated with a secretive cult with altered health seeking behavior.

Instantaneous R sensitively described real-time shifts of the incidence captured within each epicurve stage (Fig. 2). The decline stage in China was reflected by a decrease in R estimates in the latter stages the outbreak and relative to the early estimates: 1.6 (95% CI 0.4–2.9) and 1.8 (95% CI 1.0–2.7) for 2- and 7-days serial interval, respectively. Superspreading events inflated R estimates seen in exponential stage that was observed in South Korea: 2.8 (95% CI 0.6–5.3) and 25.6 (95% CI 3.0–48.2) for 2- and 7-days serial interval, respectively. Distinctive disease control was instituted in Singapore enabling it to remain in the index stage while Japan was moving to the takeoff stage characterized by increased R estimates 3.6 (95% CI 0.4–7.3) 2.2 (95% CI 1.3–3.0) for 2- and 7-days serial interval, respectively. The R estimates overlapped for all exemplar country outbreak stages in the two serial interval scenarios, suggesting that the transmission could be as short as 2 days. These estimates were relatively lower than previously reported, bringing to light the possibility of transmission during the incubation period that is associated with rapidly expanding outbreaks, which was being observed in many European countries at this time during the pandemic.

**Figure 3:**
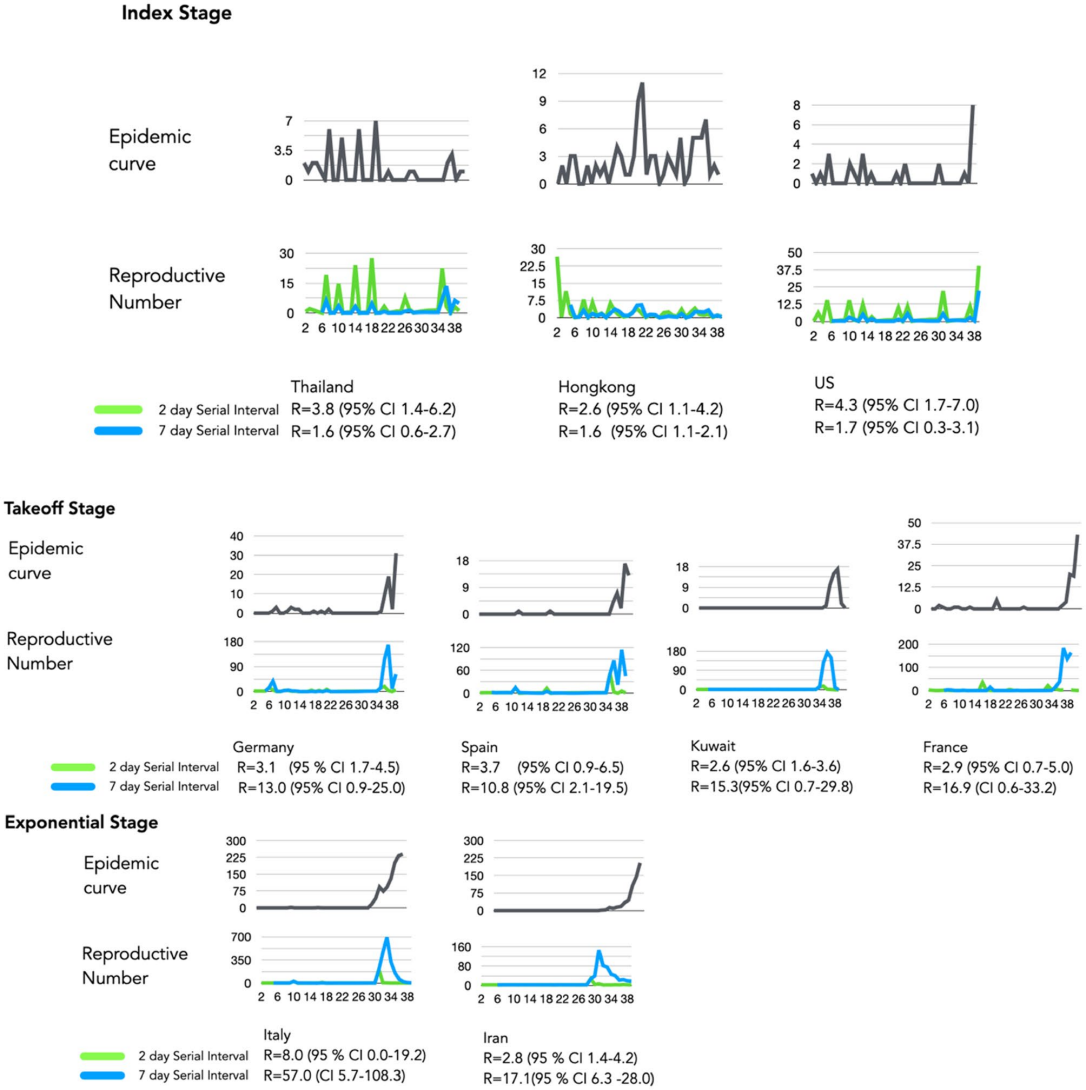
Epicurve estimates with different serial intervals. Panel Index represents Epicurves and instantaneous R values for index stage countries using 2- and 7-day serial interval. Panel takeoff Global dynamics of SARS-CoV-2 using instantaneous estimate of reproductive number with 2-day serial interval. Under preincubation period infectivity scenario, globally increasing R > 2. Italy’s R = 8 is highest due to late detection of infection clusters. This higher R estimate is due to a huge bump in cases combined with diagnostic gap of low-level incidence. The same surge dynamics is seen in South Korea. Global dynamics of SARS-CoV-2 using instantaneous estimate of reproductive number with 7-day serial interval. Italy’s R value inflates to 57 with the 7-day serial interval assumption and overlaps with the lower threshold of 2-day serial interval R estimate. This estimation depicts a decreasing pattern for countries multiple introductions like Singapore, Hong Kong.

Low case detection of COVID-19 was observed in representative countries in the index stage with R values < 2 that was attributed to effective social distancing (i.e. Hong Kong) or under detection for countries with limited testing (i.e. United States) (Fig. 3—index). Sustained local transmission occurred in five countries that were progressing into the takeoff stage (Japan, Germany, Spain, Kuwait and France) by R values > 2 (Fig. 3 takeoff). The magnitude of spread was apparent with relatively higher R estimates (> 10) in Italy, Iran and South Korea, which demonstrated sudden surges in incidence due to prior undetected clusters of cases (Fig. 3). This substantially increased instantaneous R estimates relative to other estimation methods but allowed a more obvious depiction of the surge of cases that precisely differentiated the takeoff stage from the exponential stage.

We further examined the association of country-specific instantaneous R estimates by comparing different local temperature ranges (tropical versus temperate) and population density of representative cities with outbreaks. The higher temperature range and population density were used for selected countries; however, no direct link was observed (Table 2). Case increases for South Korea were largely associated with an outbreak among a secretive religious group Shinsheonji (73% cases of COVID-19 in South Korea), located mainly in Daegu with a lower population density 883/km^2^ as compared to the rest of the areas with an outbreak^25^ and may explains the outbreak expansion early in the epicurve rather than the area’s population density. While most representative countries (Table 2) have cooler temperatures (10–6 °C), Singapore’s higher temperatures indicated that local transmission occurred at higher temperatures and suggests that temperature shifts will not likely change transmission. The temperature and population density did not explain changes in the epicurve. This led us to hypothesize that the viral genomic variation underpinned changes in the epicurve in each country.

**Table 2:** Epidemiological parameters and instantaneous R estimates.

Country	Reproductive number (R)	Temperature (°C) during outbreak	Population density (people/km^2^)	Interpretation in consideration of the epidemiological curve
Singapore	3.3	32	8136	Imported cases, limited local transmission
France	2.9	10	4300	Imported, local transmission > 1–2 month
Italy	8	10	7200	Imported cases, local transmission > 1 month
United States	4.3	9	8444	Imported cases, local transmission > 2 month
South Korea	2.8	6	883	Imported cases, local transmission > 1–2 month

The population density for South Korea is based on Daegu where 75% of the cases are reported.

**Figure 4:**
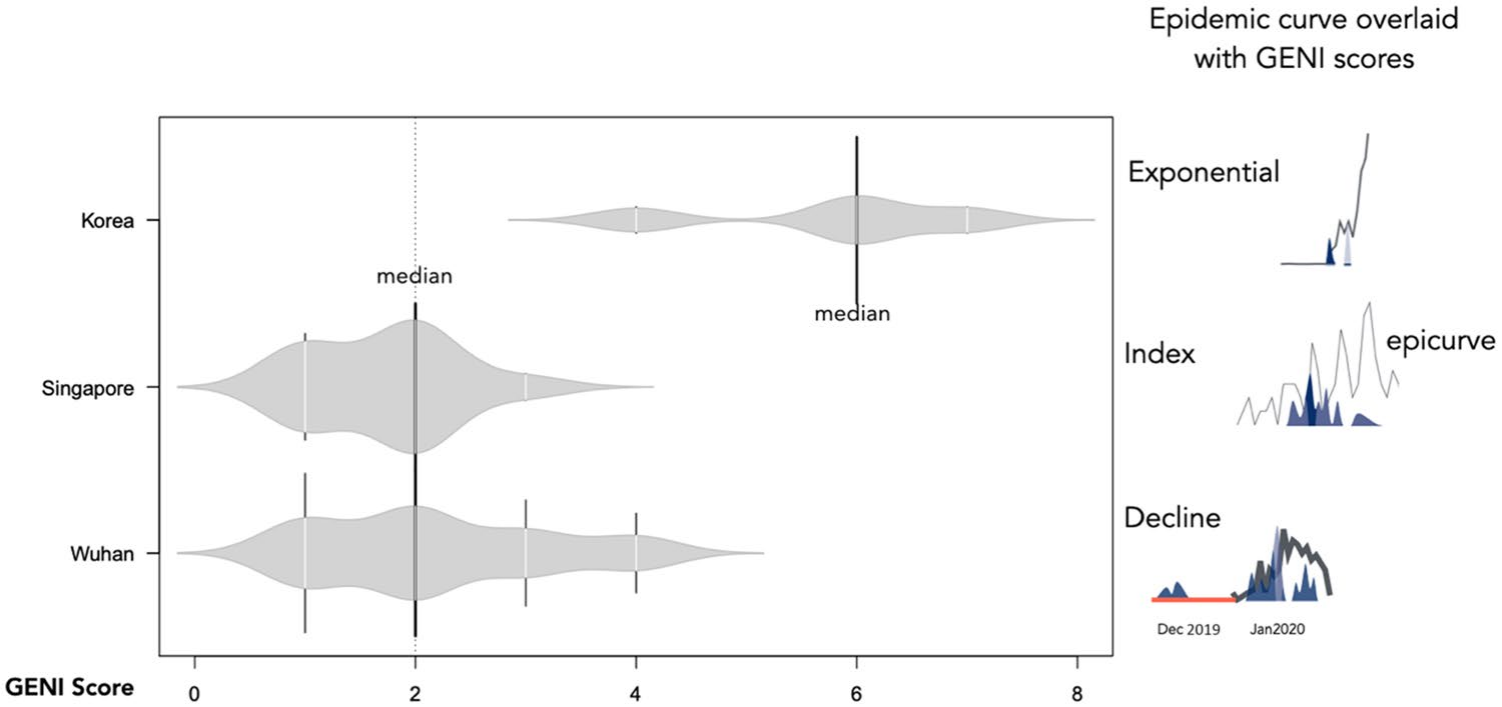
Relationship of pathogen genome identity (GENI) score with the temporal signal along the epidemic curve. Local transmission is captured by virus mutation as expressed in GENI score values. GENI scores of SARS-CoV-2 isolates are relative to Wuhan reference strain Wuhan-Hu-1 NC_045512.2. The red line in the China epicurve represents the time before an outbreak was determined yet genome sequences were circulating. The blue shaded curves indicate GENE scores directly overlaid with the outbreak curve. The dotted line represents the common point in time as a reference for visualization. The GENI score and epicurve show similarity except in China as the outbreak advanced to takeoff and exponential the GENI score increased while in the index stage example of Singapore the outbreak was contained and the GENI score remained < 2.

We determined the relationship of epicurve stage with viral genetic variation using a metric that merges absolute genome variation with the rate of genome change to create the GENI score. This approach anchored viral genome diversity with the rate of evolution for SARS-CoV-2 to create an index that is comparable between countries and progression of the outbreak. To examine how the viral genome diversity was associated with the epicurve stages we first examined the index stage (Singapore) and the exponential (South Korea). Integration of GENI scores successfully distinguished the index and exponential stages (Fig. 4). An increase in the GENI score was associated with the exponential stage at a median score = 4, suggesting that the viral diversity and rate of mutation was directly proportional to case increases during this stage. Singapore (index stage) had a GENI score = 2. This was found in multiple time points during the outbreak, where multiple mutation events were directly associated with an increase in cases. While China was in the decline stage the retrospective association with R, cases, and the GENI score provided longitudinal evidence of multiple case expansions with viral mutation events. This observation was especially clear early in the epicurve and indicated that SARS-CoV-2 was circulating in China at least 1 month prior to the official declaration of the outbreak (Fig. 4). Merging these estimates provided evidence that repeated viral mutations indicated a change in the epicurve. These metrics were associated at each time point over 3 months, in three countries, and in three different outbreak stages. This finding is useful in integrating virus genome diversity and evolution rate into assessment of outbreak status. The approach successfully replicated the observation in viral movement between countries and within a country when the epicurve was combined into a triad with instantaneous R estimates. The proportionality of GENI scores with the epicurve stage indicated the stage of outbreak as well as determining the outbreak status (Table 3).

**Table 3:** Relationship of Pathogen Genome Identity (GENI) score derived from mutational difference from the index genome (Wuhan isolate of SARS-CoV-2 or cluster isolate reference from multiple outbreak regions outside of territory).

Equivalent Pathogen Genome Identity (GENI) score for SARS-CoV-2	Clinical interpretation and epidemiological inference	Note
0–2	No difference from index case isolate genome or reference, imported case if there is no prior report, indicative of acute transmission < 1 month	Reference genome is primarily earliest isolate available
3–4	Recent local transmission (average 1–2 months) if there are no prior report of cases	Subsequent outbreak clusters can serve as sources of introduction hence near neighbor reference has to be selected to generate an accurate GENI score
> 4	Sustained local transmission (greater than 2 months) if there is are no prior report of cases	Subsequent outbreak clusters can serve as sources of introduction hence near neighbor reference has to be selected to generate an accurate GENI score

**Figure 5:**
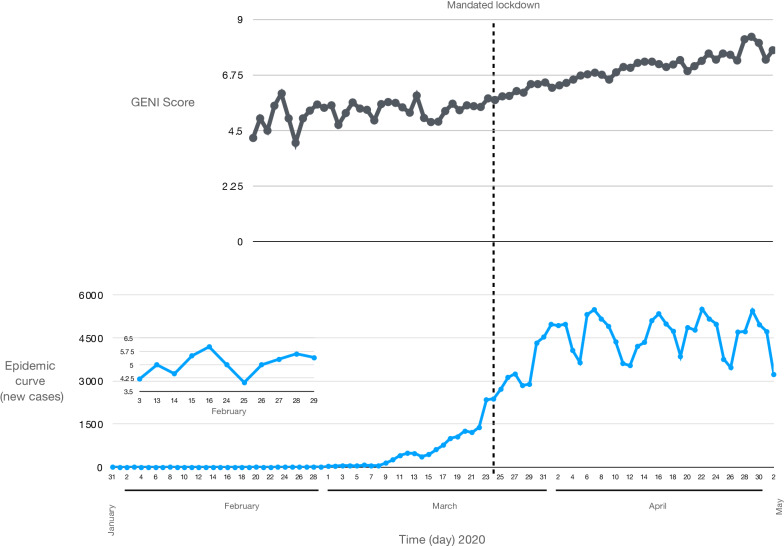
The GENI Score derived using 20,000 13,419 SARS-CoV-2 sequences from the United Kingdom (top) along with the corresponding epicurve. The inset epicurve displays the low level of cases in February indicting the outbreak was in the index phase. A high initial GENI score suggests cryptic viral transmission while a consistent GENI score indicates an increase in transmission as the pandemic progresses. This also indicates continued mutations increase viral genome diversification. While the epicurve varied after lockdown the GENI score consistently increased demonstrating continued production of genetic variation.

Further examination of this approach was done using genomes and epidemiology curves coupled to SNP variation, not lineage variation, from February to April 2020, which captured documented surges in outbreaks that were aligned with the GENI score and the newly emerging SNP variants in the UK. This analysis led to further validation that genomic variation was occurring even during lockdown that was aimed at reducing the outbreak and was predictive of recurring surges in infections using > 20,000 genomes (Fig. 5). Low numbers of new cases were observed (Fig. 5 inset) was associated with a variable GENI score (February 2020). As the cases surged in April 2020 the GENI score rose at a constant rate indicating that the genomic variation was increasing as cases were increasing. Instituting a government lockdown aimed to reduce exposure did cause variable changes in the outbreak curve it had no effect on the GENI score, which continued to rise indicating that when exposure occurred the virus was readily able to infect the person. This suggests that the underlying causes of new cases have two components—viral genome variation (evolution) and individual exposure. With this concept in mind, it can explain ‘superspreading’ events based on the continued genome evolution to maintain or expand host range that readily infect people that form large groups to quickly lead to new cases. Demonstration of this repeated observation using a longitudinal analysis with > 13,000 genomes and hundreds of cases lends extremely strong support to the notion that measuring allelic diversity is predictive of higher transmission and it will be observed when the appropriate conditions in large groups or exposure using outbreak curves. However, additional work is needed to specifically indicate the exact mutations that will initiate new cases more quickly, as demonstrated with emergence of the B.1.1.7 lineage in late 2020 within the UK and quickly spread globally.

**Figure 6:**
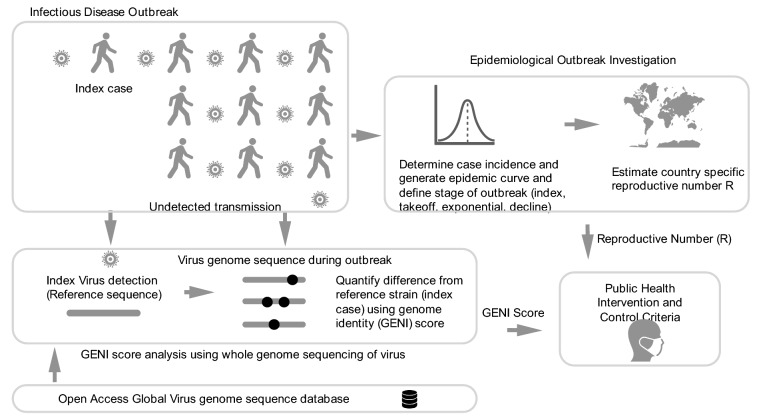
Integration of genomic and classical epidemiology for outbreak investigation. The foundation of epidemiology is the accurate and timely reporting of cases which enable the calculation of the number. Genomic Identity (GENI) score is formulated from genomic data of pathogens to differentiate imported cases versus local transmission and measure time of cryptic spread. Together these two epidemic values deliver insight that can be directly used for making decision criteria for public health intervention.

This study demonstrated an advancement of how to use population genomics using SNP variation (i.e. the underlying genetic cause of emerging variants) in an infectious disease, particularly when the mutation rate is fast and the genome diversity of the population is large, such as SARS-CoV-2. GENI scores provided a missing element of evidence that defined how to estimate new cases approximately 2–5 days before they appeared. GENI score estimation accuracy increases with analysis of large numbers of genomes (i.e. populations of genomes and populations of SNPs) and from different global locations as demonstrated (Fig. 5). Consequently, a framework to merge epidemiology and population genomics was derived from this study as a method to systematically integrate molecular epidemiology into public health (Fig. 6). It required dynamic measurements be taken for R and surveillance efforts to determine WGS for each virus. Ideally, each case would have multiple WGS as the disease progressed but this was not available. Using this triad of measurements accurately and quickly provided insight to measure outbreak progress but also provided an evidence-based method to judge intervention effectiveness.

## Discussion

Public health response is proportional to the severity and transmission dynamics of an infectious disease outbreak. This requires epidemiological metrics that can be used as decision criteria, and ideally, they can be used to assess impact of the intervention. In this work we determined that R was more dynamic in the SARS-CoV-2 pandemic than previously appreciated among the countries examined (Figs. 2, 3). This was observed in part because we used instantaneous R but also because this estimate was not collapsed into a single number but rather used as a daily estimate. The instantaneous R estimation with a serial interval of 2 was extremely sensitive to shifts in the epicurve during the index phase (Figs. 2, 3). Singapore was an excellent example of effectively controlling and containing the SARS-CoV-2 outbreak in spite of multiple mutation or multiple introduction events. They previously designated a response system called Dorscon (Disease Outbreak Response System Condition)^26^ providing a systematic approach to control, which seemed to effectively control transmission so that they did not moved beyond the index phase. In contrast, other countries in this phase were poised to move into the takeoff phase (Fig. 3). The transition into the takeoff phase was accompanied by a transition from a 2-day serial interval to a 7-day serial interval determine shifts in the epicurve.

Gaps in testing created a challenge in accurately defining the epicurve status early in the pandemic. To address this diagnostic limitation, while estimates of R alone is insightful in retrospect, they alone lacked robust predictive value present in this study. To overcome this limitation, we merged GENI estimates based on WGS variation via determination of SNP to the single initial reference strain that originated in Wuhan and the mutation rate via SNP determination over time with the epicurve and R to provide a predictive triad of measurement that resulted in insight that accurately refined case expansion (Fig. 4). Each phase of the outbreak was categorized with mutations that were associated with new cases in established outbreaks. The merged evidence indicated that China had circulating virus at least 1 month prior to the recognized outbreak (Fig. 4). Independent of the phase framework, merging GENI scores with the epicurve found new cases in the same timeframe as new sequence variants emerged. Previous studies where the relationship of genomic diversity with epidemic severity (i.e. R) found no clear link^11^. However, by merging instantaneous R, the epicurve stage, SNP variant emergence, and the GENI index we determined that a link does exist for each country examined by genome variant (i.e. SNP) and not the lineage, highlighting that this framework is also applicable to emerging variant detection and attention to the outbreak dynamic. This approach resulted in a direct prediction of outbreak dynamics and genomic mutations as well as the mutation rate by individual viral genomes and not the lineage aggregate.

The GENI index provided a basis to examine imported cases or locally spreading, both of which were addressed in this current work using established metric—R and novel integration of WGS to define changes in the sequence that were directly predictive of increases in cases. This approach leads to an epidemiological framework that is scientifically robust and at the same time can convey complex biological properties to enable an efficient characterization of an outbreak in combination. Transforming complex pathogen characteristics were accessible to the public health and medical fields using the GENI score as a complete merged information set with other characteristics of the outbreak.

Previous outbreaks, such as Ebola, employed state of the art analysis using phylodynamics that is anchored on the genetic evolution^16^. Inference, such as time to most recent common ancestor, allowed estimation of outbreak origin, population size, and R—yet this was not integrated into the outbreak dynamics and stage of advancement in the outbreak. This type of analysis is possible because genomic sequences carry temporal signals and when used in context with samples collected longitudinally, previous divergence can be determined, which has been used to do source tracking. However, the GENI score includes these signals and expands their use by merging them with the outbreak dynamic using the population genome variation as well as the mutation rate to provide an index related to the epicurve—one that was directly predictive of new cases—opposed to the genealogy of the virus.

This approach is not limited to viruses. Another recent example, in a bacterial setting, was the cholerae outbreak in Haiti wherein the phylogenetic analysis resolved the origin of the pathogen^27^. However, for this analysis to succeed, a substantial genome sequence database, of isolates collected across time and geographic location, was needed to enable placement in a phylogenetic context^28, 29^. As outbreaks are bound to happen in the future, investment in cataloguing the genomic space of pathogens is even more important than previously appreciated so that populations of appropriate size can be examined as systematically examined in bacteria^30, 31^. It is critical to obtain COVID-19 sequences from humans as well as other animals that have zoonotic potential. This was demonstrated previously with zoonotic *Campylobacter* species^32, 33^ that enabled disease in a variety of host species. Creating sequence repositories for pathogens is critical and underway for various pathogens^31^ as well as SARS-CoV-2^21^.

Small samples were used initially because the work was done as the pandemic unfolded (Fig. 2) that resulted in using small numbers of genomes. The work was repeated as the outbreak expanded with 20,000 genomes with the same outcome—SNP variant emergence leads to new outbreaks (Fig. 5). Comparing these two observations directly indicates that SNPs accurately predict new outbreak clusters and do not suffer from the population sampling bias that is created when the lineage is used.

Prior work forewarned the flaw of being overly dependent on early estimates of R alone^34^. By having the most accurate possible information for a dynamic metric and taking into account the complex dynamics that factor in the calculation of R along with merging this the WGS and mutation rates of the pathogen a robust and insightful method to assess outbreak dynamics was created in this study. Demonstration of the value of using population scale genome analysis was done with > 20,000 SARS-CoV-2 genomes from the UK country-wide sequencing program was leveraged to merge with the case reports in a 3-month period (Fig. 6). This is the first integration of allelic variation determination to create a framework beyond lineage definition. The longitudinal nature of this created a repeated measures paradigm with the GENI index and the outbreak curve that provides validation of this approach. February 2020 was the index phase followed by the takeoff phase in March 2020. The GENI index continued to increase even when social interventions were instituted—providing evidence that multiple components of transmission are at play: (1) genome variation that increase infectivity, (2) opportunity for transmission (group gatherings), (3) specific alleles emerge that manifest in low levels (index phase) and proceed to the takeoff phase when the opportunity allows.

Openness and data sharing of incidence reports and sequences at an unprecedented scale is being done in this pandemic and it is paying rewards^35^, as demonstrated in this work. Leveraging shared resources opens unexpected collaboration and avenues for applying relevant bioinformatic and disease modelling opportunities across the scientific community to solve global public health problems very quickly. Based on this approach, we propose a systematic framework to merge epidemiology and genomics that was defined and validated in this work (Fig. 6). The advantage of an evidence-based approach is the utility of WGS and surveillance that can be used to predict locations for new cases or used to quantitatively examine intervention effectiveness to control new cases and reduce the exact allele that underpins specific disease clusters and outbreaks.

## Conclusion

This study integrated population genomics into epidemiological methods to provide a framework for molecular epidemiology. Specifically, this study demonstrated epicurves, instantaneous R estimates, and GENI scores for SARS-CoV-2 are useful as pandemic metrics and in combination are a robust method. It was demonstrated that the pandemic is poised to become larger and that mutation will be associated with the increase in cases. Exemplar outbreaks, such as Singapore, found increases in cases with viral mutations that were effectively controlled. However, other outbreaks had expanding R estimates during the outbreak, as well as numerous viral mutation events. Use of epicurve stages, instantaneous R estimates, and GENI provided a robust and accurate framework to monitor outbreak progression to different stages with direct association between cases and increases in each metric.

## Supplementary Information

Supplementary Information 1.

Supplementary Information 2.

Supplementary Information 3.

## Data Availability

All data generated or analyzed during this study are included in this published article and the genome sequences were in the public domain previously.

## Abbreviations

SARS-CoV-2: Severe acute respiratory syndrome coronavirus 2
SNP: Single nucleotide polymorphism
CI: Confidence interval
Dorscon: Disease Outbreak Response System Condition
EBOV: Ebola virus
MERS-CoV: Middle East respiratory syndrome coronavirus
R: Reproductive number
WGS: Whole genome sequence
GENI: Genome identity
WHO: World Health Organization
